# The extent and nature of children’s involvement in food practice research: a scoping review of qualitative studies

**DOI:** 10.1017/S1368980023001891

**Published:** 2023-12

**Authors:** Sophie Wright-Pedersen, Helen Vidgen, Foluke Abigail Badejo, Danielle Gallegos

**Affiliations:** 1 Centre for Childhood Nutrition Research, Faculty of Health, Queensland University of Technology (QUT), 62 Graham Street, South Brisbane, QLD 4101, Australia; 2 School of Exercise and Nutrition Sciences, Faculty of Health, Queensland University of Technology (QUT), 149 Victoria Park Road, Kelvin Grove, QLD 4059, Australia

**Keywords:** Participation, Food practice, Scoping review, Nutrition, Children, Qualitative

## Abstract

**Objective:**

Aligning with the United Nations Convention on the Rights of the Child, amplification of children’s voice in food practice research aims to inform initiatives that cater to children’s needs and thus improve nutritional outcomes. The aim of this study was to describe children’s (aged 6–11 years) involvement across qualitative research investigating their food practice perspectives.

**Design:**

A scoping review was conducted according to Preferred Reporting Items for Systematic Reviews and Meta-Analyses for Scoping Reviews (PRISMA-ScR). Six electronic databases were searched up until March 2023 (Cochrane, CINAHL, Embase, ERIC, Medline and PsychInfo). The Wellcome Framework for young people’s involvement in health research guided data extraction. Data were described according to inclusiveness, geography, food-related study topic, research stage and method, and child involvement.

**Results:**

The search identified 120 peer-reviewed studies (134 papers). Active participation was only seen within research implementation stages (i.e. data collection (*n* 134), analysis (*n* 31), dissemination (*n* 9) and re-design (*n* 7)). More passive forms of participation were identified in research design stages (i.e. agenda setting, resourcing and design). Studies that utilised participatory research methodologies and developmentally appropriate and engaging methods (e.g. PhotoVoice) saw more active participation by children.

**Conclusion:**

This review identified a lack of opportunities for children’s active participation in all stages of food practice research. Without a radical shift towards providing these opportunities, food and nutrition initiatives, policies or further research that do not meet the needs of children’s food-related worlds will continue to be developed. Instead, researchers and their institutions need to advocate for and, where possible, provide voluntary opportunities for children to actively participate in food practice research.

The importance of children achieving a nutritionally adequate diet should not be understated as it is pivotal for growth and development enabling children to meet their full physical, cognitive and socioemotional potential^(1)^. Consequently, in order to impact on nutritional health, research is regularly undertaken into children’s food practices. Food practices span food-related activities of *planning, procurement and storage, preparation, consumption,* and *post-consumption* as guided by previous food practice^(2,3)^ and food literacy^(4)^ work. As a result, children’s food practices are openly and regularly monitored, critiqued and subsequently governed, particularly with regard to nutritional quality.

Most investigations into children’s food practices are from the viewpoint of adults such as parents, teachers or other professionals^(5,6)^. Children’s food practices and the determinants of their nutritional health are, therefore, drawn from experimental or observational studies from predominantly adult researcher perspectives^(5,6)^. These investigations are then used as the evidence to inform the development and implementation of a range of strategies, policies and programmes across several settings that aim to modify and govern children’s food^(7,8)^. Increasingly, there is ample evidence for involving target groups in research, in this case children, to provide more contextually relevant insights into their unique worlds. The insights can, in turn, shape more appropriately tailored strategies that are more likely to garner intended outcomes^(9,10)^.

This child-centred approach is informed by the new sociology of the child which shifts the conceptualisation of children away from passive objects to be acted upon, towards competent, capable and active subjects with influence over the construction of their own social worlds, as well as that of adults^(11–14)^. There is an additional emphasis on the diversity of childhood experiences, thus giving all children, regardless of their age, the opportunity to provide unique insights of their contextualised lived experiences. This has resulted in frameworks, such as the Wellcome Framework^(10)^, being developed to ascertain how children from a diverse array of backgrounds are involved in research. Children’s insights offer valuable perspectives of how their life worlds are constructed by them and by other actors surrounding them^(11,14–17)^. This reconceptualisation is aligned with the widely ratified United Nations Convention on the Rights of the Child (UNCRC)^(18)^ which has cemented the rights of children, regardless of their age, in having an active voice in decisions affecting their lives, including their health and wellbeing. The focus on children’s human rights recognises the power differentials that subjugate children’s voices and thus excludes them from dialogue and decisions that directly influence them^(15)^. Children’s meaningful and active participation in research has consequently begun to gain momentum, particularly in sociology and early years education, revealing positive outcomes for research, children themselves, child–adult working relationships, and their communities^(19,20)^.

For example, research designed for children’s active participation has revealed that it may cater more to children’s experiences and needs, have improved recruitment, retention and engagement of children within research, and produce richer data through improved researcher–participant rapport and more accurate ‘interpretation’ of results compared to that conducted solely by adults^(9,10,21)^. Documented benefits for children included increased self-efficacy, resilience and a sense of empowerment; skill development in research, leadership, communication, presentation and teamwork; and improved health knowledge, possibly translating into improved health outcomes^(9,10,20)^. Providing children with these platforms for active participation can facilitate improved child–adult working relationships through mutual appreciation of each other’s perspectives and experiences^(9)^. Shared decision-making may also prevent misunderstandings, disagreements or resistance to change^(9)^. Further, through these new skills and opportunities children may gain access to powerbrokers who influence policy and practices^(21)^. Benefits to the wider communities in which children are involved may include increased trust and awareness of research outcomes, and overall, a more civically engaged child population^(9,10)^.

Thus, children’s involvement within health research as active subjects rather than passive objects is vital for aligning with the UNCRC and realising the positive outcomes seen in other disciplines^(11,15,16,22)^. As described by Ergler^(23)^, active participation involves negotiations between children and adults across various research stages, whereas passive participation sees children’s involvement typically limited to the data collection phase.

Actively engaging children within research to explore factors affecting their food practices and thus nutritional wellbeing could lead to policies and initiatives that are more appropriately and contextually tailored towards their needs^(9)^. Notably, children are generally considered ‘less developed’ than adolescents and therefore less likely to be involved in research processes^(19)^. Considering the new sociology of the child, however, methods that are tailored towards children’s communication styles, instead of adult-dominant tools, may facilitate their involvement in research^(19,20)^. However, the extent and nature of children’s involvement within food practice research is unknown. Therefore, the aim of this review was to describe primary school-aged children’s involvement (as per Ergler’s definition^(24)^) across qualitative research examining their perspectives of factors affecting their everyday food practices and the methods used to perform this research. A secondary objective of the review was to reflect on the results and discuss how children’s active participation in food and nutrition research may be pragmatically implemented with consideration to existing research structures. In doing so, this review may guide best practice in child-centred food and nutrition work.

## Methods

The JBI methodology for scoping reviews^(24)^ and the Preferred Reporting Items for Systematic Reviews and Meta-Analyses extension for Scoping Reviews (PRISMA-ScR)^(25)^ were used to guide the review process. As a review of existing literature, ethical approval was not required.

### Eligibility criteria

Peer-reviewed journal articles were eligible if they (1) explored children’s (with an identifiable age of 6–11 years) perspectives of factors affecting food practices, (2) were based in a country ranked in the first thirty according to the Human Development Index (HDI; current as of 2020^(26)^), and (3) included distinguishable qualitative data attributable to a child of 6–11 years old. Primary school-aged children were chosen as the target group as they are generally considered ‘less developed’ than adolescents and therefore less likely to be involved in research processes^(19)^. Additionally, previous reviews of older age groups’ involvement in health research were identified^(27–29)^. For the purpose of this review, the term ‘food practices’ was guided by previous food practice^(2,3)^ and food literacy^(4)^ work to describe food-related activities that span planning, procurement and storage, preparation, consumption, and post-consumption of food. While World Bank classifications of countries by income is commonly used, the HDI was chosen as the defining contextual criteria as it is an objective measure of life expectancy, education levels and standard of living as per gross national income, thus providing a comprehensive conceptualisation of relative advantage inclusive of income^(30)^. Further, the HDI was used as a limiting factor to refine the scope of the review, based on being contextually relevant to the authors’ country with similar national infrastructure and institutions for children. Only studies published in English were included.

Papers were excluded if they (1) specifically sampled patient participants with diagnoses that impacted on dietary intake such as clinical eating disorders, diagnoses that restricted foods or influenced food beliefs, or in which participants were fed solely using enteral or parenteral nutrition, (2) did not report specifically on the factors that affect food practices, (3) primarily focused on alcohol consumption or nutritional supplementation, (4) were non-peer reviewed or unpublished data, (5) did not include primary data, (6) were based on quantitative research methods including the use of self-report or researcher-administered surveys, including those analysing data from open-ended questions, or (7) reported during or post-intervention analysis/evaluation of short-term nutrition-related programmes as these may not be representative of children’s everyday food practices. Additionally, recent literature reviews were identified that had already explored children’s involvement in health interventions^(19,31,32)^. Refer to online Supplementary File 1 for further detail on exclusion criteria.

### Search strategy

A preliminary search of Medline, the Cochrane Database of Systematic Reviews and PROSPERO was conducted on 26 May 2021, and no current or underway systematic reviews or scoping reviews on the topic were identified. An initial limited search of Medline and CINAHL was undertaken to identify articles on the topic. The text words contained in the titles and abstracts of relevant articles and the index terms used to describe the articles were used to develop a full search strategy for Medline (refer to online Supplementary File 2). The search strategy, including all identified keywords and index terms, was adapted for Cochrane Library, CINAHL, Embase, ERIC, Medline and PsychInfo databases and run in June 2021, again in February 2022, and in March 2023.

### Study selection

Following each search, all identified citations were collated and uploaded into referencing software EndNote 20 (https://endnote.com/) and duplicates were removed. Remaining citations were uploaded into Rayyan (http://rayyan.qcri.org), an online literature review data management system, for further refinement. The selection of relevant articles was pilot-tested by all authors with a random sample of twenty-five titles/abstracts screened using the eligibility criteria. Once 75 % agreement was met, title and abstract screening was undertaken by the first author (S.W.P) for assessment against the exclusion criteria. A sample of excluded results were verified by a second reviewer (D.G. or H.V. or F.A.B.) with high levels of agreement.

**Fig. 1:**
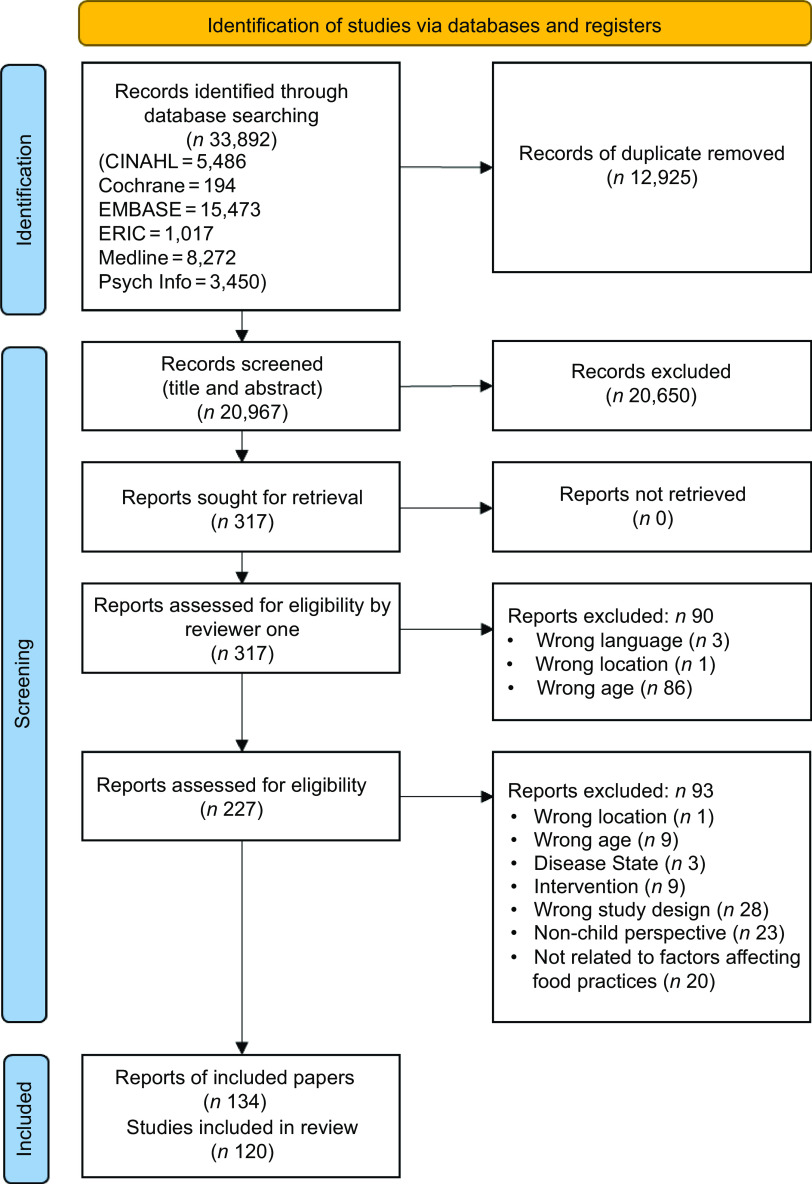
Flow diagram of the sources of evidence selection process using the Preferred Reporting Items extension for Systematic Reviews and Meta-Analyses for Scoping Reviews (PRISMA-ScR)^(26)^

Full text of potentially relevant sources were retrieved and were assessed in detail against language, geographic location and age criteria (online Supplementary File 1, Rounds A-C) by the first author. Due to the clearly defined parameters of these exclusion criteria, one author (S.W.P) was able to exclude articles. The full texts of remaining citations were assessed in detail against all inclusion criteria by the first author and a second reviewer (D.G. or H.V. or F.A.B.). Any disagreements were resolved by consensus or through involvement of a third reviewer (D.G. or H.V.). Publications that met the eligibility criteria were included in the scoping review. The full process is outlined in the PRISMA-ScR flow diagram^(25)^, as shown in Fig. 1.

### Data extraction

The authors created a data extraction matrix table which was guided by the Wellcome Framework for young people’s involvement in health research^(10)^. The Wellcome Framework was chosen as it was developed to facilitate research with young people, and frames involvement was through five explicit categories of inclusiveness, geography, health topic, research stage and the level of involvement^(10)^.

For this review, inclusiveness was extracted based on participant demographics; geography was defined as the country and study setting (i.e. school and community-based); health topic was defined as the type of food and/or nutrition topic papers focused on (extracted from the aim of the paper); research stage followed the Wellcome Framework’s agenda setting, funding, design, data collection, data analysis, dissemination, with ‘re-design’ as an additional component; and the level of children’s involvement was extracted based on what was described in studies guided by Ergler’s^(23)^ interpretation of active and passive participation. All authors piloted the extraction table on four sources to ensure appropriateness, and that all relevant results were extracted. Once consensus was reached, data extraction was performed by the first author (S.W.P) on included papers using the finalised matrix.

### Data analysis

The extent of children’s involvement was guided by Ergler’s^(23)^ interpretation of active and passive participation. This was described against the six research stages identified by the Wellcome Framework^(10)^, including the research methods used in each stage. This analysis was overlaid with the type of food and/or nutrition topic being investigated (health topic), the research setting (geography) and participant demographics (inclusiveness) to complete the Wellcome Framework^(10)^.

## Results

### Search strategy results

Database searches yielded 33 892 publications, with 20 967 remaining after de-duplication. Title and abstract screening resulted in 20 650 records being excluded, leaving 317 records sought for full-text review. A final 134 papers were included in the scoping review, of these papers, 120 were individual studies (see Fig. 1). Data extraction results for all 134 papers are available as a separate dataset^(33)^.

### Description of included studies

#### Inclusiveness

Papers (*n* 134) were published between the years 1995 and 2023, with only 7 % of papers published prior to 2007, and 11 % published from 2022 to March 2023. A majority of studies conducted solely with children (71 %), that is, they did not include adults. Only 35 % of studies included children aged 7 years or younger. Gender/sex was reported by most studies (86 %), predominantly incorporating a mix of boys and girls (73 % of total studies). Socio-economic status was reported by 79 % of studies and spanned a diversity of backgrounds using proxy indicators from the household in which the child lived (e.g. parental income, occupation and education attainment; and/or postcode of where the child lived), school (e.g. proportion of students eligible for free school meals or government support; socio-economic status indices based on attending children; and/or national indices for postcode of the school) and area levels (e.g. national indices for postcode of the community). Cultural background was reported by sixty-nine studies (58 %), a majority reported the inclusion of children from a mix of cultural backgrounds (42 %).

#### Geography (setting)

Children were usually recruited via school settings (63 %). Studies were largely conducted in this setting (55 %) followed by community settings (35 %; including twelve explicitly detailing methods within the home), with twelve studies (10 %) being conducted between the school and community. Since 2022, an increase in online qualitative methods (e.g. videoconferencing^(34,35)^, online blogging^(36)^ and YouTube video analysis^(37)^) was witnessed. A large number of studies were conducted in the USA (29 %), followed by the UK (21 %), Australia (12 %), Canada (8 %) and New Zealand (6 %).

### Food and/or nutrition topics investigated

Due to the complex and interrelated nature of food practices, the authors were unable to extract the specific aspects of food practices within each study as this was beyond the scope of this review. For example, although a paper may have focused on ‘cooking’ as a food practice, ‘procurement’, ‘consumption’ and other food practices were invariably brought up by children. Instead, the food and nutrition topics under investigation as per the aim of each paper were thematically analysed. The types of food and nutrition topics investigated varied from more focused, singular topics to broader, whole systems or wider concept investigations. Singular topics were typically focused on the intrapersonal determinants of food practices (i.e. children’s taste preferences, food knowledge and skills). In a variety of studies, investigations expanded to social and cultural influences (i.e. peers, family, schools and media), with others exploring wider socio-political scapes including food availability and socio-economic determinants (i.e. food security, dietary acculturation and food environments). Studies where children communicated a broad array of food practice determinants (i.e. spanning from intrapersonal to socio-political) generally utilised broader exploratory questions or explored concepts themselves (e.g. the concept of health) rather than specifically focusing on one type of food or adult-constructed concept (i.e. not just ‘healthy’ food).

### The nature of children’s involvement

**Fig. 2:**
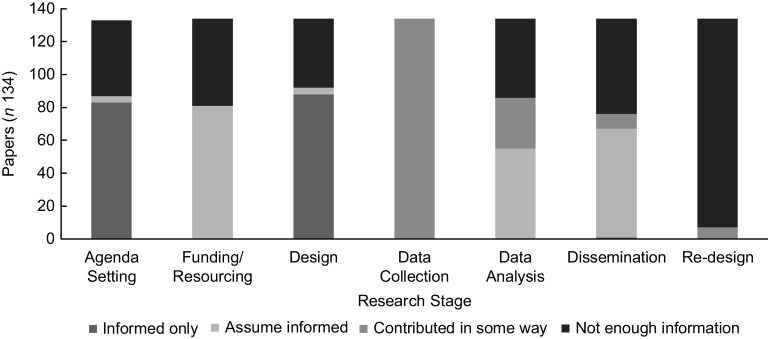
Children’s level of involvement by research stages for papers included in review (*n* 134)

Overall, children were more actively involved in research implementation stages (i.e. data collection, analysis, dissemination and re-design), as shown in Fig. 2. Notably, three studies^(38–40)^ signalled elements of children playing a dominant role in research implementation stages. All three of these studies employed participatory methodologies, with Jennings *et al.* ^(39)^ and Kovacic *et al.* ^(40)^ using community-based participatory research and Clausen *et al.* ^(38)^ participatory action research.

#### Agenda setting, funding and research design

For these three research stages, the nature of children’s involvement in food practice research mainly revolved around children being informed of the research topic *(agenda setting)* and how the research would be conducted *(research design)* via informed consent/assent procedures. Where consent/assent procedures were conducted with children, parental/guardian consent was also obtained. *Funding and resourcing* information was assumed to be included in these procedures, although no research paper explicitly mentioned this. Through employing these consent/assent processes, studies indicated that children understood the research project and why they were being asked to be involved. Additionally, these processes indicated that children had been provided the opportunity and information to volunteer or decline involvement prior to, during or after the research. Two studies reported piloting tools^(36)^ or conducting cognitive interviews^(41)^ with children prior to research implementation. However, limited details were provided as to children’s involvement in final decision-making of adaptions following these processes, rather, tools were seemingly tested upon children and then adapted by adults.

Greater detail in the reporting of informed consent/assent procedures made it easier to confirm that children had been informed. Examples of these included detailing the use of developmentally appropriate information resources^(42–45)^ and presentations^(43,45,46)^, providing children with information packs after receiving developmentally appropriate information^(43,45)^ and/or only after children had volunteered^(45,47)^, and re-visiting information and gaining child assent prior to each study component^(43,48)^.

Within a sub-set of papers (*n* 47), there was a lack of explicit informed consent or assent procedures conducted with children, indicating that they may not have volunteered to be part of the study. Instead, consent was usually gained from parents or guardians only, with one instance of parental opt-out procedures^(49)^ and one example of school-level approval only^(50)^. In some instances, ambiguous wording was used meaning that verification of informed consent became difficult, for example, ‘parents were assured of the anonymity of their children’s data’^(51)^. Children’s level of involvement could also not be confirmed when terms such as ‘families’^(52)^ or ‘all stakeholders’^(53)^ were used.

In some studies, it seemed that children were not informed about the study prior to their involvement indicating that they potentially had limited or no understanding of why they were engaging in activities or how their information would be used in this food-related research^(54,55)^. In some cases, children were randomly selected by adults (e.g. school teachers) to be involved in the study^(56–58)^. Further, limited disclosure practices meant children (and adult caregivers) may not have been fully informed of the study intent^(59,60)^ or researcher presence^(61,62)^. For example, in one study the researcher was introduced as a ‘volunteer’^(62)^.

#### Data collection

Children were involved in the data collection phase of all included research papers. More passive forms of data collection involved children providing information via focus groups or interviews or completing researcher-led tasks. Here, children gave information according to researcher-constructed questions or instructions but were not given decision-making power. More active forms of data collection were seen when children produced drawings^(41,63–70)^, maps^(71–74)^, photos^(36,38–40,75–82)^, videos^(37)^ and/or diaries^(83)^ where they were more engaged in producing data. Typically, in the case of photo-generation (generally via PhotoVoice methods), children were given control over what would form the locus of analysis in the research. Notably, for the one study where videos were produced by children^(37)^, data for the research were collated retrospectively via already uploaded videos on YouTube. This meant that children had not initially created videos for research purposes. Two studies outlined how children were trained to actively conduct data collection, both via PhotoVoice procedures^(39,79)^. In both cases, children were trained in camera use and ethics of photography by either older youth co-researchers (high school students)^(79)^ or adult researchers^(39)^.

#### Data analysis

The nature of children’s participation in analysing data produced from food practice research typically involved consulting them on their interpretations of data collected either in a group or individually. Again, this was mainly performed when children produced drawings, maps, photos and/or diaries. Multiple studies reported member-checking opportunities for child participants either during^(42,84–86)^ or after^(35,36,62)^ the data collection phase. To perform member-checking during data collection, researchers summarised responses and asked for children’s confirmation^(42,84–86)^. Methods used for member-checking after data collection involved researchers thematically analysing responses and then asking children to confirm or add to these themes^(35,36,62)^.

#### Dissemination and knowledge translation

Children were seen to participate in the *dissemination* stage via a range of methods in nine papers. In some cases, children chose their own pseudonyms^(66,87–89)^, or papers specifically mentioned children’s consent to disseminate data^(38,39)^. In more active ways, children were involved in poster development^(40,82)^ and presentation delivery^(38,40)^. Further, information about study findings being reported back to child participants strengthened the confidence of children’s research involvement^(43)^.

#### Re-design

Consultation of children on their experience of participating in the research was the only method identified within the *re-design* stage where children were involved^(38,40,75,80,82,90)^.

## Discussion

To our knowledge, this is the first review describing the nature and extent of children’s involvement in investigations of food practices. In doing so, the findings of this research can be used to inform future food and nutrition research conducted with children. Research in this area is still in its infancy, although promisingly 120 studies met the inclusion criteria with most papers published in the last 15 years suggesting that there may be momentum building for children’s participation in food- and nutrition-related research. However, the results of this review showcased that the way in which children are provided with opportunities to actively participate in food practice research are limited. Researchers from other disciplines have begun to realise the ample benefits from providing opportunities for children to actively participate within research, including positive child health outcomes^(9,10)^. Therefore, by encouraging greater participation by children in food and nutrition research, public health nutritionists may be able to incorporate child-informed knowledge of food practices into food and nutrition initiatives. These initiatives would subsequently better cater to children’s food practice needs and likely produce intended nutrition outcomes^(9,10)^.

Overall, studies included a broad range of children from varying age, gender/sex, socio-economic status and cultural identities. School sites dominated study recruitment and implementation, similar to previous reviews in the broader health research context^(19)^. Although there are benefits to schools as research sites, power imbalances may be greater in this adult-led setting compared to more child-adapted settings, for example, youth centres, and should be considered when conducting research^(91,92)^.

Results from this review revealed that children had more passive, rather than active, involvement in food practice research, particularly in planning stages (i.e. agenda setting, funding/resourcing and design), a finding echoed by other researchers within health and other disciplines^(10,92)^. The *data collection* stage saw the most active child participation, where in some cases adults were seen to relinquish power to share decision-making with children^(93)^. Again, as reflected by Ergler^(23)^ and others^(10,92)^, this finding is common in research with children and may indicate that this is the easiest or most convenient stage to involve children in the research process compared to other research stages. However, greater passive participation of children in food practice research may also be influenced by children’s level of desire to participate when given a choice. Thus, more active forms of research may not always equate to ‘better’ forms of participation^(91)^. The *dissemination* stage also saw children co-constructing and co-delivering outputs, although not to the extent seen in the *data collection* stage. Aligning with Ergler’s^(23)^ interpretation of active participation, it has been argued that children can only be counted as co-researchers when they are partners at every step of the research process^(94)^, which none of the included studies managed to achieve. Additionally, even though children were seen to participate at more active levels in research implementation stages, this was performed under adult censorship. For example, in the *data analysis* stage, children were more likely to verify results rather than be involved in the analysis of raw data. Therefore, for this review, children acted as research supporters, informed of the research process and voluntarily providing valuable data, but not co-researching. The pragmatics and feasibility of research, however, including the influence of researchers and research structures, such as time constraints and complexities of research ethics boards, should be considered^(29,92)^ and are discussed below and in Table 1.

**Table 1:** Considerations for future research exploring children’s perspectives of food- and nutrition-related concepts

Objective	Strategy
Aim for the most active participation desired by children across all research stages as practically and safely possible.	• Ensure that children are, at the very least, adequately and appropriately *informed* of the research process and how their data will be used; and that children’s *assent/consent* is actively gained before the research has begun and iteratively throughout the research process in a way that they feel comfortable to say ‘no’. • Recruitment processes should consider children’s ability to *voluntarily choose* to participate without risk of coercion by other adults or peers, or broadly, from institutional pressure. • Seek out ways to *equitably* allow for active participation opportunities within and/or across studies. This should consider intersections across minority population groups. • *Conduct research in ways that actively engage children*, for example, opening up research agendas to be more exploratory, establishing child advisory groups, training children as co-researchers and/or incorporating developmentally appropriate methods such as PhotoVoice. Participatory research methodologies may be useful in guiding the research design. • Consider *creating relationships with localised agencies* and individuals who have access to and contextual understandings of children within their communities to provide insight into possible inequities and appropriate ways of implementing research and engaging diverse population groups. Establishing long-term relationships within communities may additionally allow for children to initiate research proposals. • Find ways to *address societal and structural barriers* to enable more meaningful and active participation of children. For example, advocating for more flexible funding models, resourcing and organisational timelines according to research needs, and continuously advocating for or providing training for other researchers to establish children’s active participation as the norm within dominant research discourse.
Continuously perform reflective practices during design and implementation of all research stages and processes.	Particularly the influence of: • Key decision-makers (i.e. is this the children themselves or others?) • Assumptions made by researchers or others • Power imbalances between children and adults, institutions and even other children • Perceived or real barriers or opportunities to processes that would allow for more active participation
Improve the reporting standards to include the extent and modes of children’s participation in research	• Publishing institutions should be equally responsible for upholding standards for consistent reporting • Consider structuring reporting standards around GRIPP2 Long Form (LF) domains^(108)^ Additionally consider and explicitly state the following: • Differentiating children from generalised population groups, that is, avoid using grouping terms such as ‘families’ • Whether or not children had an active role at each research stage, detail of this involvement and responsible party for this decision (i.e. children’s level of voluntary choice)

In regard to extending children’s more active participation to food practice research planning stages and across the life cycle of research, Kellett^(92)^ importantly points out that children must be trained in doing so, just as adult researchers are. Once training has been undertaken, children may be able to meaningfully participate in research governance and design based on their own agendas^(92)^. Within this review, only two studies incorporated training for children within the data collection phase for PhotoVoice processes^(39,79)^. For adults to support training processes across all research stages and subsequently facilitate opportunities for children’s active participation in food and nutrition research, adults must have trust in relinquishing power to be ‘supporters’ rather than ‘managers’^(92)^. For example, when researchers within this review relinquished control to children in research implementation stages, children could expand upon and adapt adult-determined agendas to match their priorities. A further step towards this may be to open up research agendas to be more exploratory so that children may be able to communicate aspects of their diverse lived food experiences viewed as most important to them.

Mechanisms found to support children’s more active participation within food practice research implementation stages were participatory research methodologies and the inclusion of developmentally appropriate resources and methods. These align with the new sociology of the child in understanding that children may have different ways of communicating than adults and their involvement in research may be limited if adult-appropriate resources and methods are solely used^(16,95,96)^. Foundationally, participatory research aims to address unequal power structures within knowledge creation though active participation of research subjects^(97)^. Therefore, this methodology may be best suited to amplify children’s voices and provide them with various degrees of control within research settings^(21)^. Performed through iterative research cycles, participatory research also has the potential to afford children greater opportunity to participate more actively in research planning and governance stages alongside co-producing or leading research implementation. Although participatory research may provide a level of insight into children’s food experiences unafforded to adult researchers, it should be implemented with a degree of caution due to the complexities of potential exposure to sensitive or traumatic information for young people^(21)^. Importantly, within all research methodologies children as the target group need to be given the same participatory opportunities as other stakeholders whilst recognising power structures at play and considering the time it takes to implement this type of research.

### Balancing ethical, idealistic and pragmatic considerations in food practice research design

The critique above highlights just how ethically, methodologically and practically complex research with children can be^(21)^. The balance between protecting children from potential research burden while attempting to amplify their voices and facilitate agency is an ongoing discussion within childhood research^(20,21)^. Table 1 outlines considerations for future research exploring children’s perspectives of food- and nutrition-related concepts.

Within the current review, children’s active participation across all stages of food practice research was not realised. This is similar to findings of other review articles of children’s involvement in the broader health arena that consistently reveal less studies achieving active participation^(19)^. However, more active forms of research may not always equate to ‘better’ forms of participation ^(91)^. For example, when children do not wish to participate in more active ways. Thus, the degree of participation that children are afforded by adults and institutions should also be considered, and how this may be limited by wider social and structural factors.

Therefore, the pragmatics of children’s participation within food practice research, complemented by an ongoing reflection as to the intended beneficence to children, must be considered for the outcomes of this review to be useful. For example, barriers to children’s participation in the included studies may have included: a lack of researcher knowledge or organisational will; a lack of resources (e.g. time and money) which may have been dictated by funding bodies, as well as research agendas with very limited flexibility to adjust to children’s food priorities; difficulty accessing children, particularly when initiating from a university setting; and research governance processes (e.g. ethics review boards) delaying research when safeguards need to be implemented^(29,92)^. Therefore, institutions and their governance structures (including research institutions) hold responsibility for the degree of participation that children are afforded, alongside food and nutrition researchers themselves. Looking to other disciplines, the Children’s Research Centre at the Open University, UK, is an exemplar of institutional dedication to empowering children to actively participate in all stages of research, largely through actively engaging children in research training, support and dissemination opportunities both within the centre and through place-based initiatives^(92)^.

The time-consuming nature of research additionally needs to be considered and how it may compete for other priority roles in children’s lives^(21,96)^. Strategies to overcome these challenges, however, are possible as exemplified by Kellett^(92)^ and the Children’s Research Centre^(92)^, who have devised a range of research training methods to be flexible to the context of children’s lives. Options include a weekly research club, integrating training within school activities, delivering over three dedicated sessions, and a 2-week intense programme that could be delivered in school holidays.

This highlights the importance for children to be given the opportunity and then supported to be involved in food-related research according to their preference^(91,92)^. This aligns with the notion of voluntary choice in research participation, which may also include participating in less active ways^(93)^. Therefore, researchers must also be vigilant about coercing or forcing children to ‘participate’ in food-related research,^(93,98)^. In fact, children appropriating, resisting, deviating from or manipulating adult-constructed research methods or even researchers themselves may be an attempt to assert their own power within research contexts^(98)^.

Finally, equity of children’s involvement is another important consideration as children and their experiences of childhood and food practices are not homogenous. Consequently, participating children should not be taken as being representative of larger groups^(21,92)^. Populations described as ‘hard-to-reach’ or marginalised are potentially less likely to engage in research due to structural barriers. Therefore, cohorts identified as potentially more marginalised should be specifically targeted and research methods tailored accordingly to ensure that these voices are not subjugated further^(21,96,99)^. Examples of targeted population groups were seen in this review, including Indigenous and First Nations^(39,41,79,86)^, new migrant^(100)^, economically disadvantaged^(85,101–104)^ and food-insecure^(105–107)^ children. Localised, place-based research processes (e.g. via youth and community centres, social housing, or remote communities) that meet diverse community needs may assist in achieving this.

### Limitations

There are limitations to this review, notably the eligibility criteria which purposefully excluded specific countries, papers written in languages other than English and specific population groups as well as the specific focus on qualitative studies. These decisions were made, however, to gain contextually relevant information and refine the scope of the review. Future reviews may additionally contribute by exploring literature from low- to middle-income countries, children aged less than 6 years and/or with children with lived experience of food-related or food-managed conditions. In line with the new sociology of the child, it was decided that to inform how to best perform research that amplifies children’s voice in food practice research, and qualitative literature would most likely reveal the most useful insights. However, further exploration of the quantitative literature may expand upon the result of this review.

Further, the subjective nature of interpretation combined with the lack of detail provided in some studies meant that assumptions were made, even after much discussion within the research team. For example, the inclusion of data analysis and dissemination information in children’s informed consent/assent procedures was assumed unless explicitly detailed. Consistent reporting of children’s involvement in food-related research would assist future assessments and provide guidance for those wishing to perform similar research. The Guidance for Reporting Involvement of Patients and the Public (GRIPP2) tool^(108)^ is an example of existing quality reporting standards in health and social research that could inform a tool for children.

### Conclusion

This scoping review has revealed the lack of opportunities for children to actively participate in food practice research, particularly in setting the research agenda, decisions regarding resourcing or the research design. Without a radical shift in how we present these opportunities, food and nutrition initiatives, policies or further research that do not meet the needs of children’s food worlds will continue to be developed and implemented.

Instead, food and nutrition researchers and their institutions need to provide voluntary opportunities for children to actively participate in food practice research. Although this can be pragmatically challenging within the confines of current research structures, food and nutrition practitioners can learn from other disciplines on ways to currently facilitate these opportunities, for example, opening up food and nutrition research agendas to be broad and able to be reshaped by children; upskilling children as researchers so that they themselves can construct and guide food practice research; and/or employing democratising methodologies such as participatory action research. These researcher-based actions may be complemented by advocacy for structural changes to reduce systemic barriers to children’s active involvement in food practice research.

By balancing these ethical, idealistic and pragmatic considerations, food practice research that seeks to actively involve children may begin to align with the UNCRC and the new sociology of the child within the current bounds of research systems and structures. This will benefit not only children’s nutritional outcomes but also the impact of this food practice research.

## Supplementary material

For supplementary material accompanying this paper visit https://doi.org/10.1017/S1368980023001891
